# Induced Opening of the Gastroesophageal Junction Occurs at a Lower Gastric Pressure in Gerd Patients and in Hiatal Hernia Subjects than in Normal Control Subjects

**DOI:** 10.1155/2010/857654

**Published:** 2010-03-18

**Authors:** Anil Vegesna, Ramashesai Besetty, Amit Kalra, Umar Farooq, Annapurna Korimilli, Keng yu Chuang, Robert Fisher, Henry Parkman, Larry Miller

**Affiliations:** Section of Gastroenterology, Department of Medicine, Temple University Hospital, Philadelphia, PA 19140, USA

## Abstract

*Purpose*. To determine intragastric pressure threshold for inducing gastroesophageal junction (GEJ) opening in normal control subjects with and without hiatal hernia, and in patients with gastroesophageal reflux disease. *Methods*. This study was performed in 13 normal volunteers, 5 volunteers with hiatal hernia, and 3 patients with gastroesophageal reflux disease. During endoscopy a pressure transducer was used to measure baseline gastric pressures. The pressure in the stomach was measured while air was insufflated into the stomach until the gastroesophageal junction opened on endoscopic view. *Results*. There were two patterns of GEJ opening in normal volunteers. The mean opening pressure for Gastroesophageal junction in normal pattern-I, normal pattern-II, hiatal hernia, and Gastroesophageal reflux patients was 11.5, 12.6, 3.4, and 1.3 mmHg, respectively. *Conclusions*. GEJ opening is induced at a significantly lower pressure in subjects with hiatal hernia and in patients with gastroesophageal reflux disease than in normal volunteers.

## 1. Introduction

Gastric distension is a trigger for gastroesophageal junction (GEJ) opening [1, 2]. To study GEJ opening in patients with gastroesophageal reflux disease (GERD), Straathof et al. used a short duration provocation test with gastric distension by air insufflation into the stomach with prolonged intraluminal recording of pressure [3]. These investigators perfused air into the stomach through a manometry catheter in two 750 mL increments. Kahrilas et al. infused intragastric air into the stomach at a rate of 15-mL/min and found an increase in the frequency of GEJ opening [4].

The purpose of the current study is to determine the intragastric pressure threshold for inducing GEJ opening in normal control subjects with and without hiatal hernia (HH), and in patients with GERD.

## 2. Material and Methods

### 2.1. Subjects

The study consisted of 13 normal volunteers (9 Men, and 4 Women with mean age of 35 ± 7 years), 5 volunteers with hiatal hernia (4 Men, and 1 Women with mean age of 36 ± 9 years) and 3 patients with GERD (3 Men with mean age of 42 ± 7 years). University Institutional Review Board approved the study protocol, and informed consent was obtained from all the participants. None of the subjects had a history of surgical manipulation of the upper gastrointestinal tract. 

Exclusion criteria for the normal subjects included any abdominal symptoms or any medication that could affect the gastroesophageal segment high-pressure zone, including the use of antacids, H2 blockers, proton pump inhibitors, prokinetic agents, erythromycin type antibiotics and anticholinergics, GERD, hiatal hernia, conditions and disorders including a history of esophagitis, abdominal pain, heartburn, regurgitation, chest pain, difficulty swallowing, pain on swallowing, dysphagia, abdominal surgery involving the stomach or esophagus, nausea or vomiting, diabetes, scleroderma, esophageal motility disorders, noncardiac chest pain, achalasia, and current pregnancy. Exclusion criteria for the GERD patients were hiatal hernia on endoscopy or any history of surgery on the esophagus or stomach. There were no dropouts in the study as this is a single visit outpatient study.

### 2.2. Endoscopic Evaluation of the Study Subjects

**Figure 1:**
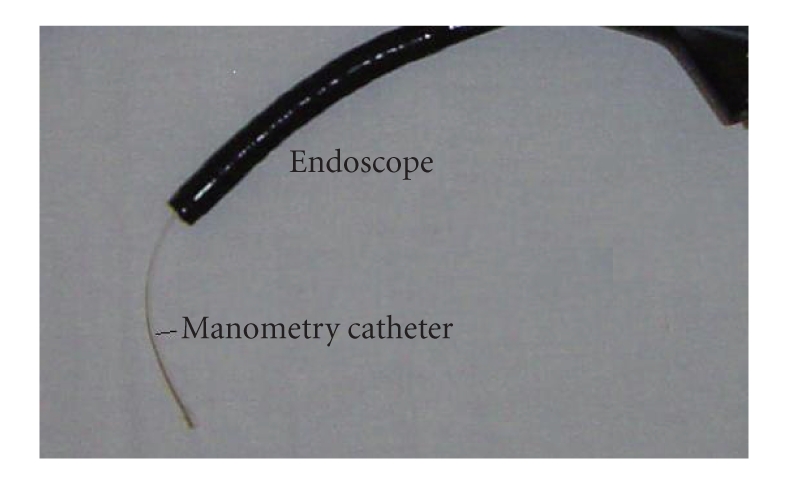
Picture of an endoscope with a manometry catheter placed through the biopsy channel.

All subjects underwent upper endoscopy after an overnight fast with an Olympus GIF H180 endoscope or OLYMPUS GIF N180 or Pentax EG endoscope. Subjects were kept in the left lateral decubitus position during the procedure. Benzocaine spray was used to anesthetize the posterior pharyngeal wall as the endoscopy was performed unsedated. Subjects were evaluated for the presence of esophagitis and for any abnormalities in stomach, and duodenum including hiatal hernia. The entire procedure was videotaped for all the subjects. After viewing the esophagus, stomach and duodenum, water perfused manometric catheter was passed through the biopsy channel of the endoscope (Figure 1). 

### 2.3. Manometric Evaluation

**Figure 2:**
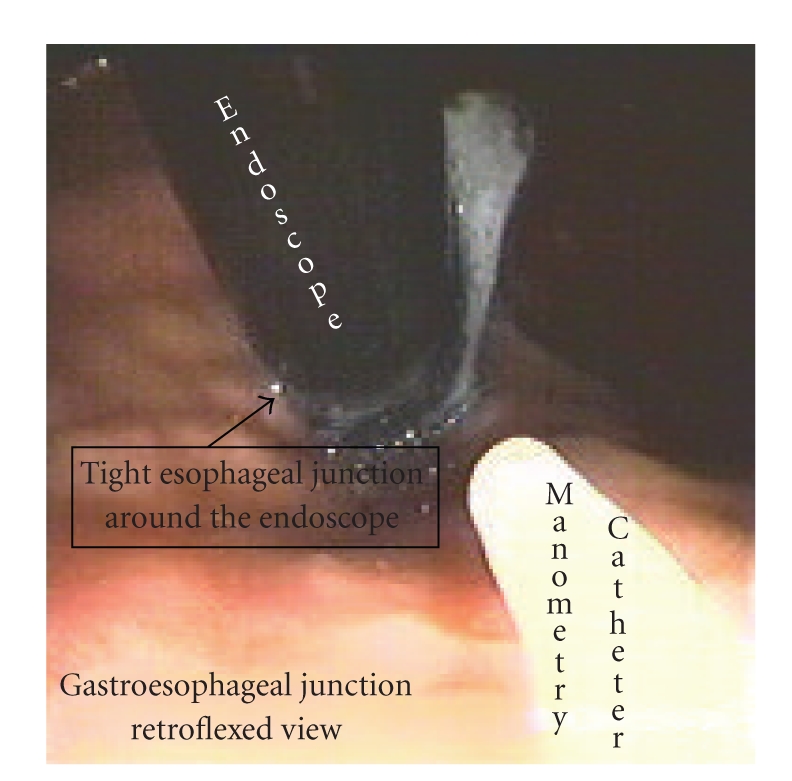
This picture shows a retroflexed view of the gastroesophageal junction in a normal volunteer. The white catheter is the manometry catheter and the black tube is the endoscope. Note that the gastroesophageal junction is tight around the endoscope prior to air insufflation into the stomach.

Manometric studies were performed using a single port water perfusion manometric catheter, which was passed through the biopsy channel of the endoscope. This manometry catheter was continuously perfused with gas-free distilled water by a low compliance pneumohydraulic capillary infusion system (Arndorfer Medical Specialties, Greendale, WI) at a rate of 0.5 mL/min. After visualizing the stomach all the air in the stomach was removed by suction through the endoscope. Any remaining air from the cardia of the stomach was removed and a baseline pressure was recorded keeping the endoscope in a retroflexed position along the lesser curvature side of the stomach (Figure 2). After recording a steady baseline pressure for few minutes air was insufflated into the stomach continuously at a constant flow through the endoscope until the gastroesophageal junction (GEJ) opened or until the subjects complained of discomfort. Between each gastric distension sequence, the stomach was kept deflated in order to measure a steady gastric baseline pressure for 15 to 30 seconds. This was repeated 5-6 times in order to exclude trials in which the pressure readings were abnormal due to abdominal contraction, belching or due to swallows. 

A dental suction device was placed in the subject's mouth to prevent accumulation and swallowing of saliva. In addition an acoustic monitor was placed on the subject's neck to assess for swallowing and burping. The whole study was recorded on a Kay elemetrics swallowing workstation (KayPENTAX, 2 Bridgewater lane, Lincoln Park, NJ). This system was used to synchronize the pressure readings to the video recording of the endoscopic procedure. During the study the subject was monitored to see if there were any swallows. As this was an unsedated study the subject was also asked to notify the team if he/she swallowed during the study. If swallows were observed, those trials were discarded and were not included in the analysis.

The studies were analyzed from recorded video images and pressure recordings on the Kay Elemetrics swallowing workstation in a blinded manner. The video images were evaluated to determine opening of the hiatus without knowledge of the pressure at that time point. Average pressure was then obtained at that time point for each individual subject for the five to six gastric distention's that were performed.

## 3. Statistical Analysis

Results are presented as means ± standard error. The variables were compared between groups using an unpaired *t*-test. For all the results, an associated probability (*P*-value) of less than.05 was considered statistically significant.

## 4. Results

Out of 18 normal subjects 5 were found to have hiatal hernia during the endoscopy. The remaining normal volunteers without hiatal hernia did not have any other abnormalities of the esophagus, stomach, or duodenum on endoscopy. Three patients with GERD and without hiatal hernia were also evaluated (3 males, average age 42 ± 7 years old). 

**Figure 3:**
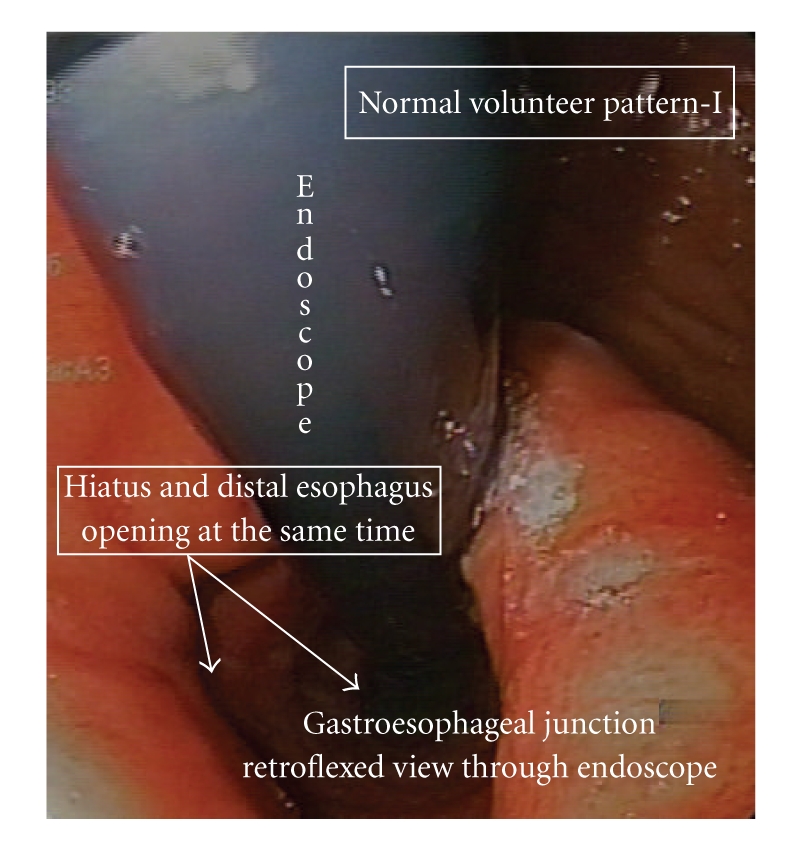
This is pattern I opening. This picture shows a retroflexed view of the gastroesophageal junction in a normal volunteer. Note that the entire distal esophagus is open around the endoscope after air insufflation into the stomach.

**Figure 4:**
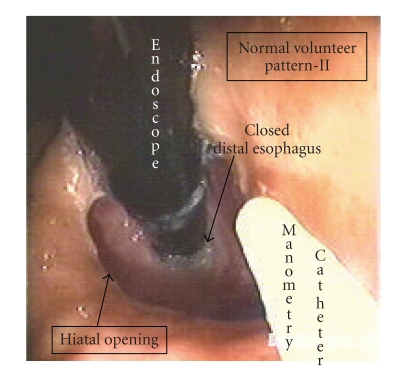
This is pattern II opening. This picture shows a retroflexed view of the gastroesophageal junction in a normal volunteer. The white catheter is the manometry catheter and the black tube is the endoscope. Note that the hiatus is open around the endoscope during air insufflation into the stomach but that the distal esophagus remains closed and tight around the endoscope. This subject did not have a hiatal hernia, and the esophagus was tight around the endoscope prior to air insufflation.

**Figure 5:**
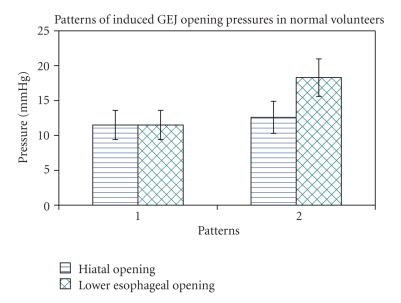
This graph shows the two different patterns of GEJ opening in normal subjects.

In the 13 normal volunteers without hiatal hernia the stomach was inflated for an average period of 26 seconds during each insufflation. Two patterns of gastro-esophageal junction opening were identified in the 13 normal volunteers. In pattern I (10 normal subjects) the hiatus slowly stretches and deforms; however the hiatus and distal esophagus opened simultaneously, allowing the expulsion of air from the stomach into the esophagus (Figure 3). The mean gastric pressure at the point of distal esophageal opening in pattern I was 11.5 ± 2.1 mmHg. After hiatal opening the gastric pressure dropped slightly by about 2-3 mmHg. In pattern II (5 normal subjects, 2 of the normal subjects had overlap between patterns I and II during multiple distension studies) the hiatus opened rapidly after insufflation of air into the stomach. Then at some time point later and at a higher pressure, the distal esophagus opened allowing for expulsion of air into the esophagus (Figure 4). The mean gastric pressure for opening in pattern II was 12.6 ± 2.3 mmHg for the hiatus and 18.3 ± 2.7 mmHg for the distal esophagus (Figure 5). The mean length of time that the hiatus remained open on endoscopic visualization after the initiation of opening was 17.1 ± 3.3 sec. for type I opening and 37.6 ± 5.0 sec. for type II opening after the initial hiatal opening.

In the 5 subjects with hiatal hernia the gastroesophageal junction opened at a mean pressure of 3.4 ± 1.3 mmHg above the gastric baseline, which is significantly lower than in the normal control subjects (*P* < .05). In hiatal hernia subjects only GEJ opening was reported since the gastric pressure component extends proximal to the hiatus.

**Figure 6:**
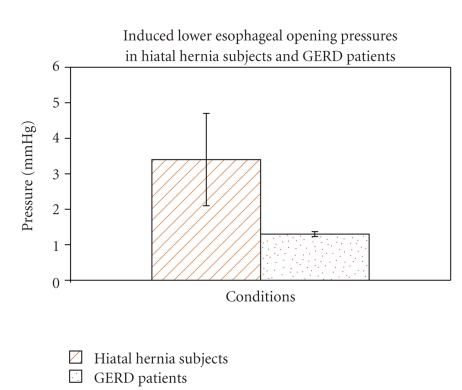
This graph shows the hiatal opening pressures of the two patterns of normal subjects, subjects with hiatal hernia and the GERD patients.

In the 3 GERD patients the mean gastric pressure at hiatal opening was 1.3 ± 0.07 mmHg which is significantly lower than in the normal control subjects without hiatal hernia (*P* < .05) (Figure 6). All the observed GERD patients have only pattern I type of GEJ opening (hiatus and distal esophagus opened simultaneously). It should be noted that an opening pressure of 1.3 ± 0.07 mmHg, measured by a water perfusion manometry system, is practically indiscernible from resting baseline pressure.

In all cases there was endoscopic evidence of esophageal body contractions after the distal body opened, in the absence of swallows, as viewed endoscopically. The time of hiatal opening recorded during swallows was usually less than 5 sec. 

## 5. Discussion

Many patients with GERD have normal resting lower esophageal sphincter pressure on manometry it has become clear that other factors must contribute to the pathogenesis of GERD and that a static measurement of lower esophageal sphincter pressure, using conventional techniques, is not a true assessment of lower esophageal sphincter function. 

The lower esophageal sphincter pressure may be abnormally low on a transient rather than a sustained basis. The mechanism of physiologic gastroesophageal reflux is most commonly attributed to transient lower esophageal sphincter relaxation [5–7]. Transient lower esophageal sphincter relaxations (TLESR) are relaxations of the gastroesophageal junction high-pressure zone, which occur in the absence of swallowing. This occurs in both normal volunteers and in GERD patients but may occur at a higher frequency and may last longer in GERD patients [6, 8, 9]. 

It is impossible to state with certainty that the GEJ openings in this study were transient lower esophageal sphincter relaxations, since no manometric catheter was placed across the GEJ. However the GEJ opening that we observed during the gastric distension had characteristics similar to the transient lower esophageal sphincter relaxations. The duration of lower esophageal sphincter (LES) relaxation is a major variable that distinguishes TLESR from swallow-induced LES relaxation. The duration of swallow-induced LES relaxations is only 6–8 seconds TLESRs last significantly longer and almost always longer than 10 seconds with virtually no overlap between the two types [10–13]. The time length of LES relaxation during swallows was less than 5 seconds in this study. In addition, in the current study we documented prolonged relaxation of the hiatus during induced GEJ opening in the normal control subjects (17–37.6 seconds). Finally a prominent after contraction is also a characteristic feature of TLESR. In all cases, in this study, there was endoscopic evidence of esophageal body contractions after the distal body opened, after the induced GEJ opening in the absence of swallows. 

Gastric distention is a potent stimulus for GEJ opening. This is not surprising given the fact that GEJ opening is the mechanism by which gas is vented from the stomach during belching [14, 15]. Approximately 15 mL of air is delivered to the stomach with each swallow [16]; without a built-in venting mechanism, uncontrolled gastrointestinal bloating would occur. In humans, a 750–1000 mL increase in gastric volume causes a fourfold increase in the rate of GEJ opening within the first 10 minutes after the increase [3]. 

We found that GEJ opening was triggered in the normal subjects with hiatal hernia and in GERD subjects without hiatal hernia at a significantly lower gastric distention threshold than in normal volunteers without hiatal hernia. Massey et al. described a similar technique. They were able to identify manometrically verified sphincter relaxation, which preceded opening of the GEJ [17].

The fact that the hiatus opens at a low pressure threshold for GEJ opening in normal volunteers with hiatal hernia may be explained by the disruption of the normal anatomy in the area of the gastroesophageal junction high-pressure zone. It is more difficult to explain the low pressure threshold for GEJ opening in the GERD patients without hiatal hernia. 

Recent evidence by Brasseur et al. indicates that the high-pressure zone at the esophageal-cardiac junction actually consists of three individual high-pressure zone components [18]. There is an extrinsic component, which is the crural diaphragm, and two intrinsic components, which consist of a superior physiologic lower esophageal sphincter and an inferior gastric sling fiber/clasp fiber complex. In recent findings by our group we demonstrated a lack of the distal intrinsic pressure profile, in GERD patients, consistent with a defect in the gastric sling/clasp fiber muscle complex previously demonstrated by Miller et al. in normal control subjects [19]. 

One possible explanation for the low pressure distension threshold for GEJ opening in GERD patients is that the absent distal pressure profile, due to the defective gastric sling/clasp muscle fibers, causes the distal portion of the high-pressure zone to distend at a lower pressure threshold. Another possible explanation is that the area of the defective gastric sling/clasp muscle fibers starts off more distended in the GERD patients than in normal subjects, due to the lack of tonic contraction from this muscle group. It is possible that the GEJ opening we observed, like the TLESR is induced by the stretch receptors in the cardia, the GEJ opening would therefore be initiated at a lower gastric distension threshold (a lower gastric volume and pressure) than in normal subjects, if these receptors were already partially stretched at rest. 

Interestingly, Schiffner [20] measured the stress to stretch ratios of this area during deglutitive inhibition with water bolus swallows to simulate a TLESR. They demonstrated that the stiffness of this area in normal control subjects and patients with GERD was statistically the same, but that the resting radius and initial opening radius were significantly larger in the GERD patients than those in the normal control subjects.

It is interesting that two patterns of hiatal opening were found in normal subjects in this study during GEJ opening. In pattern I the entire lower esophagus appeared to open simultaneously. This is consistent with the findings of other investigators who have reported that all of the components of the high-pressure zone relax at once during GEJ opening. However, in pattern II the hiatus appeared to relax initially and then the rest of the lower esophagus relaxed at some time point later and at a higher intragastric pressure. 

The finding of the second pattern of GEJ opening suggests that there may actually be differential relaxation of the intrinsic high-pressure zone components of the distal esophagus. Pattern II is consistent with the initial relaxation of the more distal intrinsic pressure profile (gastric sling/clasp fiber muscle complex). A later relaxation of the proximal pressure profile (the physiologic lower esophageal sphincter and/or crural diaphragm) was then seen to relax at a higher intragastric pressure. This pattern would have been hidden from prior investigators using a Dent Sleeve, since this device measures only the highest pressure recorded within the high-pressure zone. 

We plan to further study GEJ opening by placing a high-resolution manometry catheter across the gastroesophageal junction of normal control subjects and patients with GERD, during constant air insufflation into the stomach. By studying this phenomenon we hope to be able to determine if all of the components of the gastroesophageal high-pressure zone relax simultaneously or if there is differential relaxation of various components as suggested in the current study.

##  Disclosure Policy 

There are no competing interests to the authors in this research.

##  Ethics Committee Approval 

The experiment was conducted with the understanding and the consent of the human subject. Temple University Institutional Review Board approved the study protocol, and informed consent was obtained from all the participants.
